# Virtual reality cricothyrotomy – a tool in medical emergency education throughout various disciplines

**DOI:** 10.1186/s12909-025-06816-5

**Published:** 2025-02-17

**Authors:** Valentin Burkhardt, Marianne Valette, Iva Speck, Omar Flayyih, Christine Huber, Angela Widder, Robert Wunderlich, Friederike Everad, Christian Offergeld, Tobias Albrecht

**Affiliations:** 1 Department of Otorhinolaryngology - Head and Neck Surgery, Faculty of Medicine, Medical Center - University of Freiburg, University of Freiburg, Killianstraße 5, 79106 Freiburg, Germany; 2 Department of Nuclear Medicine, Faculty of Medicine, Medical Center - University of Freiburg, University of Freiburg, Hugstetterstraße 55, 79106 Freiburg, Germany; 3 Dean’s Office for Human Medicine, Faculty of Medicine, Medical Center - University of Freiburg, University of Freiburg, Breisacher Straße 153, 79110 Freiburg, Germany; 4 Department of Anesthesiology and Intensive Care Medicine, University Hospital Tübingen, Tübingen, Germany; 5 Department of Otorhinolaryngology, Head and Neck Surgery, University Hospital Tübingen, Tübingen, Germany; 6 Medical Faculty Tübingen, TIME - Tübingen Institute for Medical Education, Tübingen, Germany

**Keywords:** Cricothyrotomy, Gamification, Education, Virtual reality, VR, Emergency medicine

## Abstract

**Objectives:**

A cricothyrotomy represents an emergency procedure that may be considered a last option for securing the airway. While fortunately rare, it is important to note that such invasive procedures must be mastered if they are to be used. Therefore, regular training is essential to gain routine. The aim of the present study was to investigate whether professional groups with different levels of experience with the procedure succeed in learning the procedure with a virtual reality trainer.

**Materials and methods:**

In a multicenter approach, 146 employees with four different professional backgrounds—otorhinolaryngologists, anesthesiologists, emergency physicians and certified nurses—were included in the study. The participants were required to complete a virtual reality (VR) cricothyrotomy scenario in three consecutive runs, and the time required and errors in the procedure were recorded. The training experience was subsequently evaluated subjectively using a questionnaire.

**Results:**

The study included 146 participants with an average age of 33 years and an average of 5 years of professional experience. The majority of participants (74%) reported an improvement in the speed of the procedure and in the procedural steps (87%). These subjective improvements were confirmed objectively by the time required for completion of the procedure and the points achieved. Gaming experience had a significant effect on both the score (*p =* 0.023) and procedure time (*p =* 0.039), whereas age and medical specialization did not. Real-life experience with cricothyrotomy had no significant effect on performance in VR.

**Conclusion:**

Virtual reality provides an effective method for training healthcare professionals in cricothyrotomy, regardless of their specialty or prior experience. The participants showed significant improvements in both the speed and accuracy of the procedure after training, regardless of their prior experience or medical background. Further research is necessary to assess the benefits of VR simulation for training cricothyrotomy in real-world procedures.

**Trial registration:**

DRKS00031736, registered on the 20th of April 2023.

**Supplementary Information:**

The online version contains supplementary material available at 10.1186/s12909-025-06816-5.

## Introduction

In life-threatening emergency situations, cricothyrotomy is used as an invasive method to secure the airway in patients experiencing respiratory distress. It is only performed in patients if orotracheal or nasotracheal intubation fails and if mask ventilation cannot be sufficiently performed [1]. Cricothyrotomy is a procedure that can be performed by a variety of healthcare professionals, either in a hospital setting, in the field, or even in combat situations [2–4].

Cricothyrotomy is a very rare procedure, which makes training residents and other medical staff difficult [3]. A lack of training leads to poorer performance of surgical procedures and, in this case, cricothyrotomy. Training often includes models or even tracheas of animals, which can help improve the technique and handling of surgical instruments. However, animal tracheas, in particular, are limited and single-use items. For ethical and financial reasons, they cannot be widely used to train all medical staff involved in cricothyrotomy. Furthermore, performing a cricothyrotomy in an isolated trachea lacks the atmosphere of an emergency situation. Therefore, we first extended the current curriculum of medical students via virtual reality (VR) cricothyrotomy [5]. Owing to promising results in the student curriculum, VR simulation was integrated into interprofessional emergency training in a second step.

Medical VR simulations include elements of gamification, such as a point system, progress bar, awards, social interaction and story lines, which can be used as valuable approaches in non-game content [6]. The use of these tools helps in the design of engaging and motivating teaching programs. Therefore, gamification is becoming increasingly important in diverse and evolving teaching landscapes, with several digital and non-digital applications already established in clinical education [7]. This concept should not be confused with offering a game for training; VR simulation rather uses gamification elements to improve learning and uses strategies of the gaming industry to maintain participants’ engagement and motivation [8].

A 2021 survey on the current status of the use of simulation-based training in the education of otorhinolaryngologists (ORLs) at a global scale revealed that the most prevalent skills acquired through simulation training among novice ORLs were tracheotomy (50.4%), emergency cricothyrotomy (48.9%), and rigid bronchoscopy (47.5%) [9]. These procedures are all part of emergency care, which highlights the importance of simulation training in the field of emergency cricothyrotomy. There is an increasing proportion of simulation training already taking place in VR. However, a VR simulation model comparable to the one used in the present study for learning and practicing cricothyrotomy has not yet been described in the literature.

Therefore, we conducted the present study to evaluate the use of our VR simulation training throughout various medical disciplines.

The different groups included ORLs, anesthesiologists, and emergency physicians, as well as nurses, ensuring a comprehensive range of experience with the procedure. This includes expertise in the day-to-day management of respiratory problems through a basic understanding of anatomical and medical principles. The aim of the division into groups was to investigate whether VR simulation can be successfully used as a teaching method for different professional groups with differing degrees of involvement in the procedure. The central question was the subjective evaluation of VR training via a questionnaire adapted from a previous study by our research group [5]. Our hypothesis proposes that using a VR simulation is applicable across all disciplines and is equally accepted as a method for acquiring cricothyrotomy skills.

## Materials and methods

### VR cricothyrotomy

The VR cricothyrotomy scenario was developed using the C# programming language in the open-source platform Unity (2023, Unity Technologies, Fan Francisco, USA) as previously described [5]. A scoring system was implemented to evaluate the participants’ performance during VR cricothyrotomy. The maximum achievable score is 100 points, reflecting an error-free execution of the procedure. Errors during the performance of cricothyrotomy result in a reduction of points, down to a score of 0 [4].

**Fig. 1:**
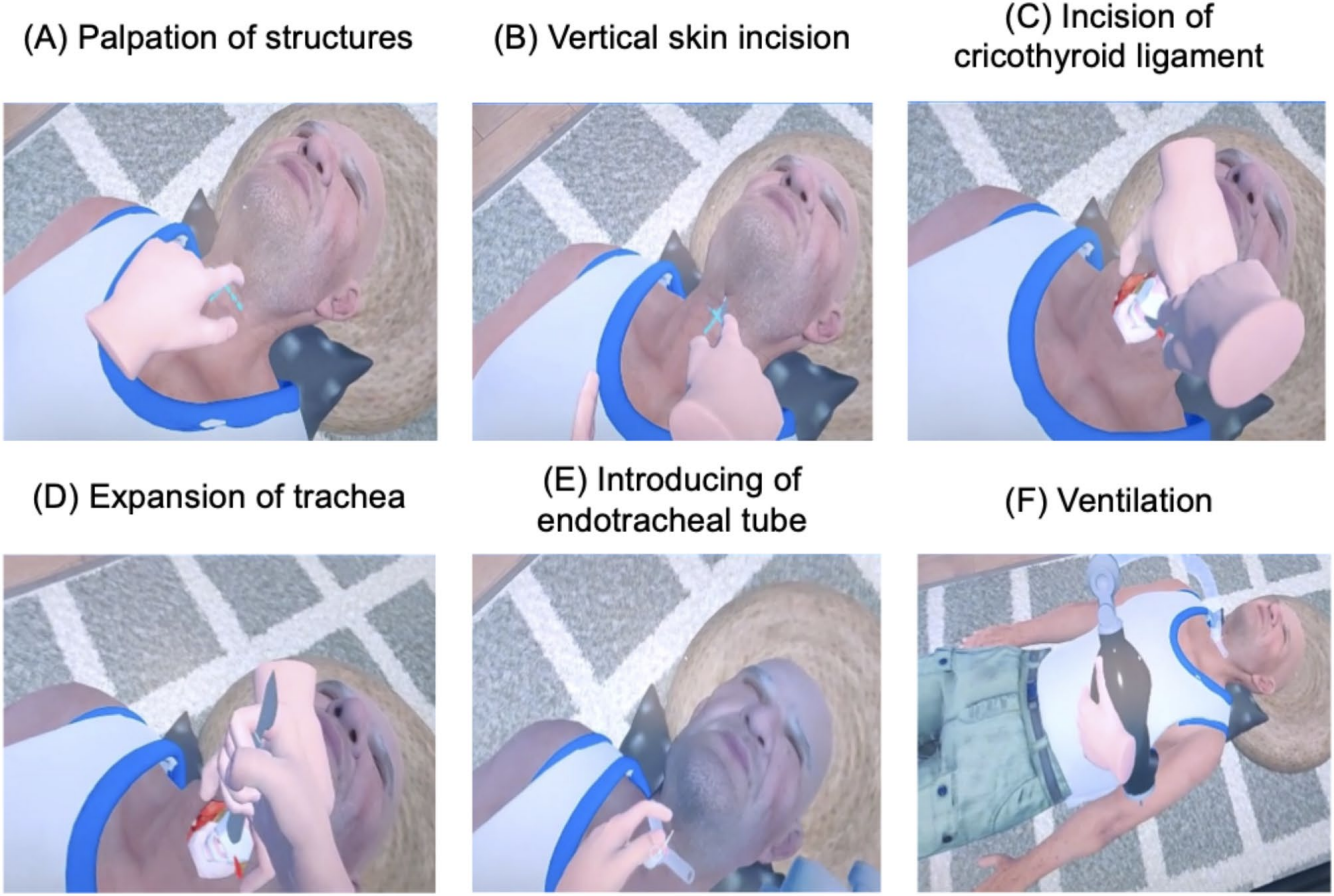
Steps of the VR cricothyrotomy scenario, adapted from Speck et al. [5]

Furthermore, the time is recorded, and the simulation ends after 120 s, as the patient is presumed to have died. Figure 1 provides a graphical illustration of the procedural steps that need to be completed within the simulation. In brief, these steps are outlined as follows: palpation of the throat, vertical skin incision, horizontal incision of the cricothyroid ligament, expansion of the trachea with the scalpel handle, introduction of the endotracheal tube, and ventilation.

Prior to the initiation of the simulation, the participants had time to familiarize themselves with the VR room and the controllers. Once the participants had become adequately oriented, they were permitted to commence the simulation.

### Study design

This multicenter, non-randomized, prospective study was conducted at the University Hospitals of Freiburg and Tübingen between July 2023 and November 2023.

Participation was voluntary, and withdrawal was possible at any time.

As part of the study, the participants first completed a theoretical training program lasting 30 min. This covered the anatomy of the neck, the management of a difficult airway, the steps of a cricothyroidotomy and the interdisciplinary management of a difficult airway.

Subsequently, each participant completed the virtual reality simulation in three consecutive sessions, with the elapsed time and resulting score for each session being recorded. Following each session, the participants were provided with feedback on their performance, including the time taken and any errors made. Following the final session, the participants completed a questionnaire to evaluate the virtual reality simulation.

Following the completion of the three sessions of VR cricothyrotomy, the participants were asked to evaluate the simulation experience using a questionnaire based on the Münster questionnaire for the evaluation of extra module role-playing games (Table 4 in supplementary material [10]),.

Members of four different medical professional groups were included: ORLs, anesthesiologists and emergency physicians, some of whom have experience with the cricothyrotomy procedure and are confronted with airway problems in their daily work. Additionally, nurses who work in an ORL ward and only assist in cricothyroidotomy were included.

### Statistical analysis

The test results were evaluated anonymously. The score achieved and the time taken to complete the task were analyzed comparatively between the study groups using the Shapiro‒Wilk test for evaluation of a normal distribution and then using the t-test or Wilcoxon rank test. In addition, the results of the questionnaire were compared across the different study groups. Based on the group distribution, ANOVA was used as a parametric test for normally distributed variables, and the Kruskal‒Wallis test was used as a nonparametric method.

Kendall-Rank-Correlation was used to calculate the correlation between Question 6 and time, as well as between Question 7 of the questionnaire and the achieved score. The primary questions (Q6 + Q7) were not adjusted for multiple testing. A *p-*value < 0.05 was considered to indicate statistical significance for all analyses. Data analysis was conducted using SPSS Statistics 29 (IBM, Armonk, New York, USA).

## Results

### Cohort analysis

A total of 146 participants were included in the study. The median age was 33 years, and the mean age was 33.39 ± 8.21 years. The participants had a median work experience of 5 years, with an average of 7.86 ± 9.58 years. No statistical significance was observed in the gender distribution, with 78 (54.17%) women and 66 (45.83%) men (*p* = 0.129, Table 1).

The participants were distributed among the professional groups as follows: 39 (26.71%) ORLs, 45 (30.82%) anesthesiologists, 30 (20.55%) emergency physicians, and 32 (21.92%) certified nurses. The distributions of work experience and gender did not differ significantly among the four groups (Table 1). A total of 12 participants in the study already performed cricothyrotomy on a patient in a real world setting and all procedures were successful: seven ORLs (17.95%), four anesthesiologists (8.89%) and one emergency physician (3.33%) (Table 2).

**Table 1:** Overview of cohort characteristics categorized by medical specialty

	Otorhinolaryngologists	Anaestesiologists	Emergency physicians	Certified nurses	*p* value
**N**	39	45	30	32	
**Age (years)**					0.007
Mean ± SD (Median)	32 ± 7 (30)	34 ± 6 (34)	34 ± 5 (33)	38 ± 13 (36)	
**Work experience (years)**					0.128
Mean ± SD (Median)	5.88 ± 6.68 (3)	5.68 ± 4.85 (5)	4.98 ± 3.05 (4.25)	15.70 ± 15.53 (13)	
**Gender**,** n (%)**					0.129
male	18 (46.2)	25 (56.8)	14 (46.7)	9 (29)	
female	21 (53.8)	19 (43.2)	16 (53.3)	22 (71)	
**Performed a cricothyrotomy before**,** n (%)**	7 (17.95)	4 (8.89)	1 (3.33)	0 (0)	0.033

### Questionnaire

**Fig. 2:**
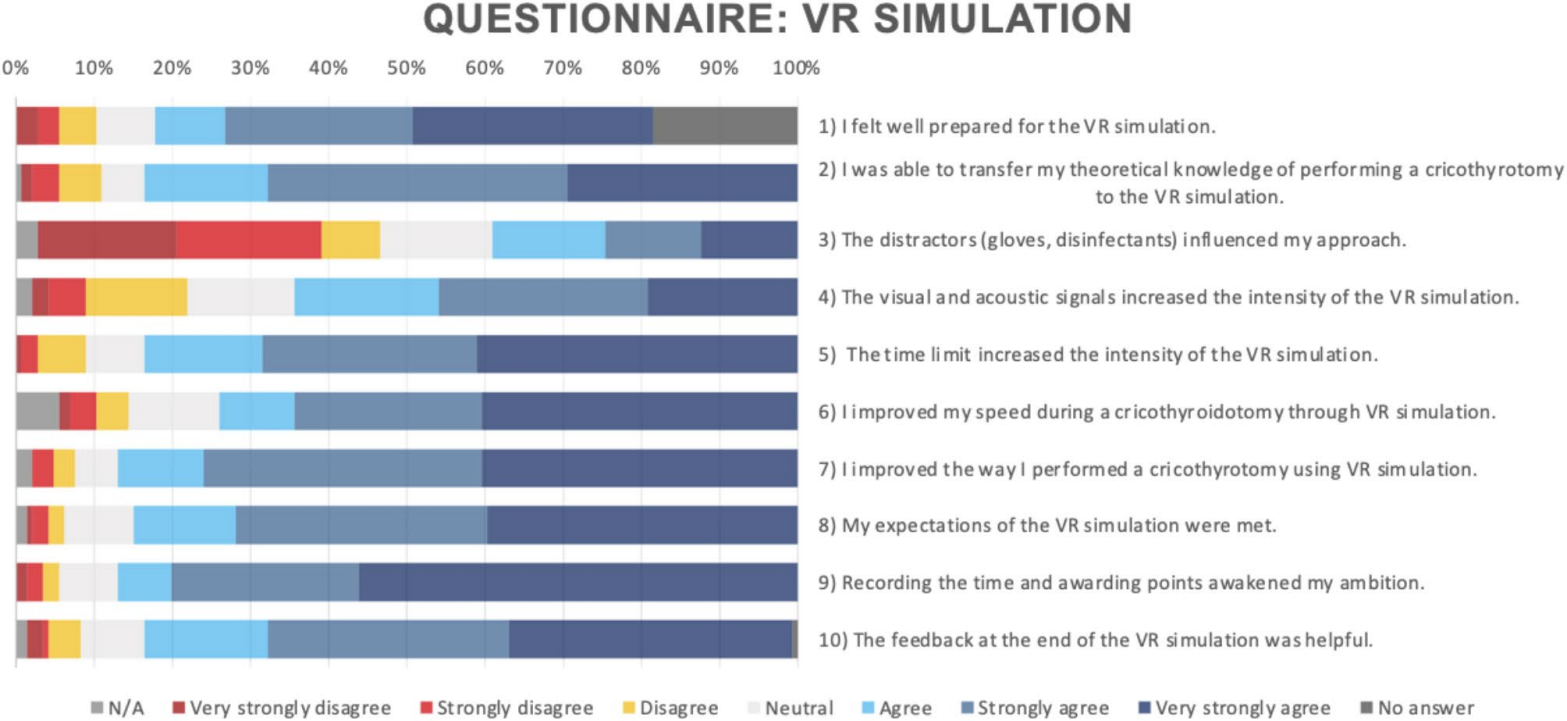
Evaluation of the questionnaire depicting answers given in the whole cohort

The results of the questionnaire were evaluated purely descriptively. 93 (78.2%) of the participants felt well prepared for the VR simulation through the previous training (Q1). A total of 122 (83.6%) participants agreed that they could apply their theoretical knowledge to the VR simulation (Q2). The distractors in the VR simulation did not lead to distraction regarding cricothyrotomy in 64 (Q3: 42.8%) subjects. The majority stated that the visual and acoustic signals (Q4: 64.4%) and the time limit (Q5: 83.6%) made the VR simulation more intense. Questions Q8 (My expectations of the VR simulation were met, 84.9% agreement), Q9 (Recording the time and awarding points awakened my ambition, 87% agreement) and Q10 (The feedback at the end of the VR simulation was helpful, 83.4% agreement) were all answered positively (Fig. 2).

Question 6 (I improved my speed during cricothyrotomy through VR simulation) was answered with “disagree” by 13 (8.9%) of the participants; 17 (11.6%) were neutral towards the statement, and 108 (74%) agreed with the question. Question 7 (I improved the way I performed a cricothyrotomy using VR simulation) was answered with “disagree” by 8 (5.5%) participants and neutral from a further 8. A total of 127 (87%) participants agreed with question 7 (Fig. 2).

The highest proportion of participants with gaming experience was found in the group of anesthesiologists (42.22%), whereas only nine certified nurses (28.12%) had such experience (*p =* 0.615, Table 2). The proportion of participants who reported experience with VR simulations was even lower. A total of 10 (25.64%) ORL participants stated that they had prior VR experience, whereas three participants in the emergency physician group (10%) did (*p =* 0.393, Table 2). Only one ORL and one certified nurse regularly played VR games.

The participants were requested to evaluate their competence in performing a cricothyrotomy before (Q16) and after (Q17) the training session. This assessment was conducted using the German school grading system, which ranges from 1 (very good) to 6 (unsatisfactory). The mean score prior to training was 3.9 ± 1.62, whereas the mean score following training was 3.1 ± 1.02. The median scores were 4 and 3, respectively. Prior to training, the group of anesthesiologists rated themselves as the most competent (mean: 2.78 ± 1.36), followed by the ORL (3.13 ± 1.25), certified nurses (mean = 4.69 ± 1.29) and emergency doctors (5.2 ± 0.93). Following the training, the ORL (2.27 ± 0.88) rated themselves the highest, followed by the certified nurses (3.0 ± 1.13), the anesthesiologists (3.2 ± 1.04), and the emergency physicians (3.43 ± 0.68). The overall rating (Q18) of the simulation was evaluated on a scale of 1–15, with 15 representing a very good rating. The mean score was 11.58 ± 2.98, with a median value of 12. The assessment of the emergency physicians was the most positive, with a mean rating of 12.37 ± 1.89, followed by the ORL (12.07 ± 1.75), the certified nurses (11.83 ± 3.74) and the anesthesiologists (10.73 ± 3.2).

The VR simulation was also rated predominantly positively within the subgroup of participants, who already performed a cricothyrotomy. The results were comparable to those of the cohort without real-life cricothyrotomy experience, as they felt well prepared for the VR simulation through the previous training (Q1: 71.4%), agreed on being able to use their theoretical knowledge (Q2: 91.7%), stating the improved intensity of the VR training through the acoustic signals (Q4: 75%), and time limit (Q5: 83.3%). Furthermore, they agreed that their expectations for the VR simulation were met (Q8: 75%), that ambitions were awakened through time and awarded points (Q9: 83.3%), and that feedback in the end was helpful (Q10: 72.7%). Questions 6 and 7 were also rated positively. Here, 75% of Q6 and 66.7% of Q7 agreed. Only the question about distractors (Q3) was rated positively less often (25%) than in the rest of the cohort.

**Table 2:** Answers to the questionnaire for questions 11 to 15 categorized by medical specialty

	Otorhinolaryngo-logists	Anaestesio-logists	Emergency physicians	Certified nurses	*p* value
**N**	39	45	30	32	
**Q11: Do you have gaming experience?**,** n (%)**					0.615
Yes	15 (38.46)	19 (42.22)	10 (33.33)	9 (28.13)	
No	24 (61.54)	26 (57.78)	20 (67.67)	23 (71.87)	
**Q12: I have experience in VR simulation**,** n (%)**					0.393
Yes	10 (25.64)	8 (17.78)	3 (10)	5 (15.63)	
No	29 (74.36)	37 (82.22)	27 (90)	27 (84.37)	
**Q13: I play VR games regularly**,** n (%)**					0.535
Yes	1 (2.56)	0 (0)	0 (0)	1 (3.13)	
No	38 (97.44)	45 (100)	30 (100)	31 (96.87)	

### Results of VR simulation

**Fig. 3:**
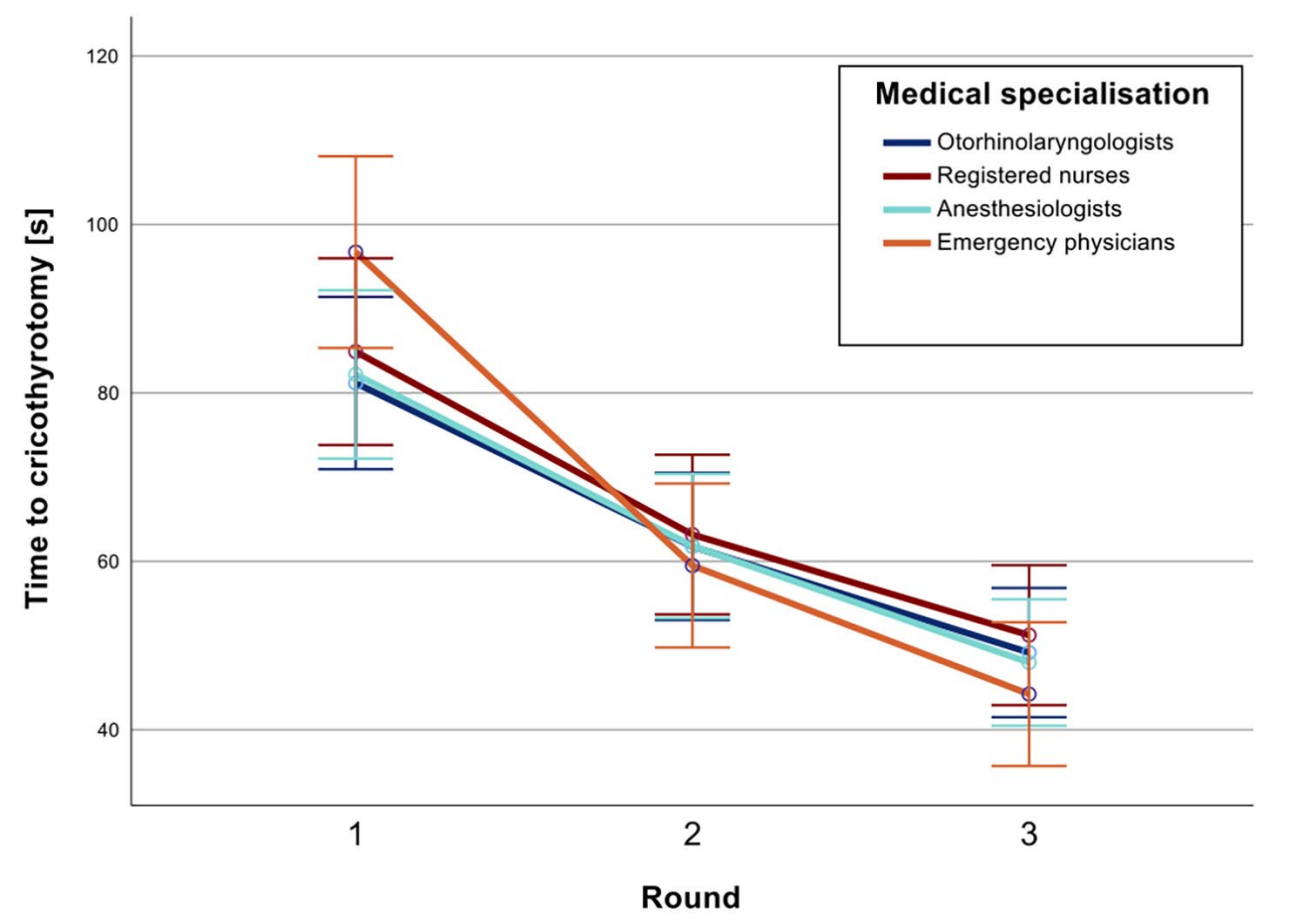
Time to complete the cricothyrotomy procedure in seconds over 3 rounds in VR simulation training. Mean value with 95% confidence interval

**Fig. 4:**
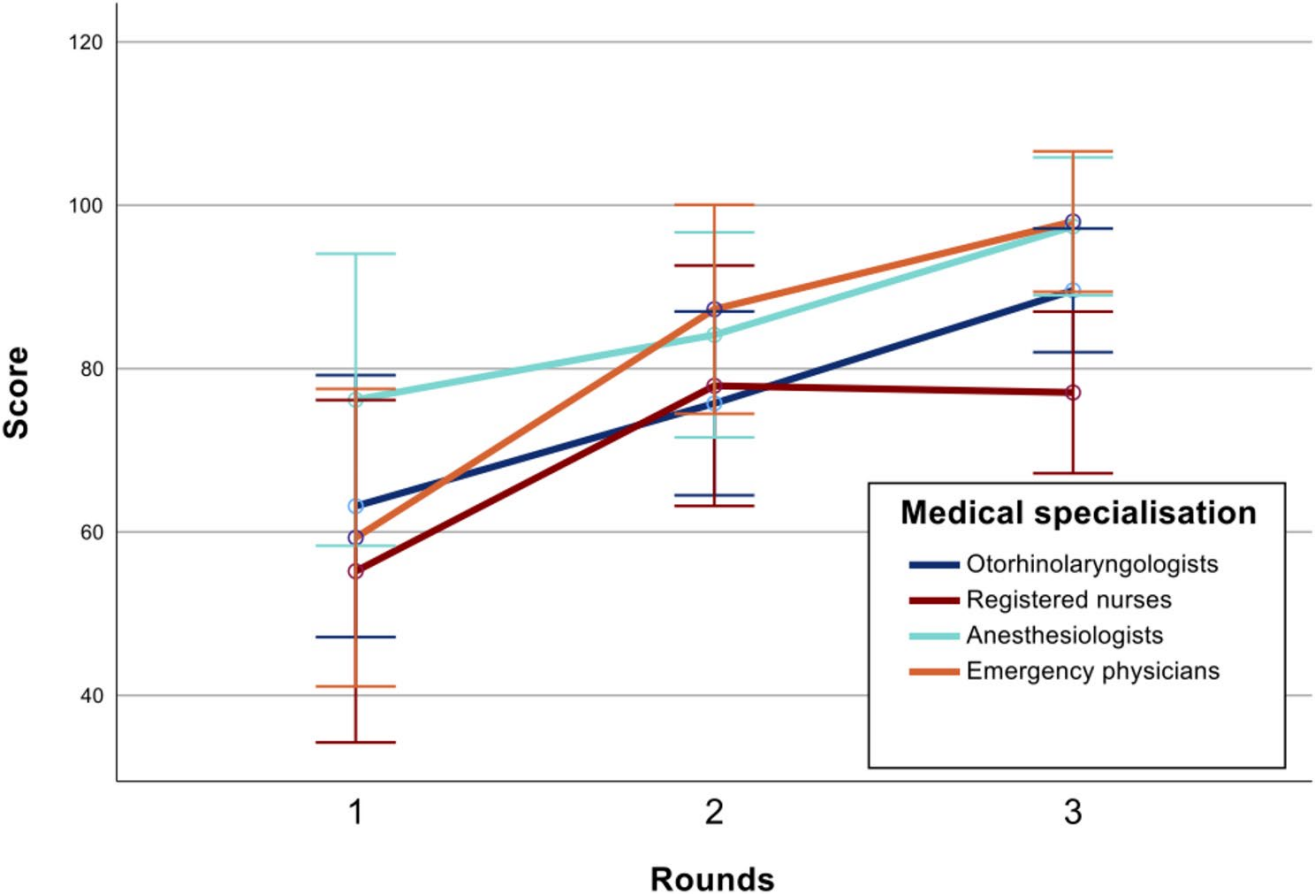
Achieved numeric score over 3 rounds of VR simulation training. Mean values with 95% confidence intervals

The participants finished the VR simulation on average in 87 ± 27 s in the first round, 62 ± 26 s in the second round and 48 ± 22 s in the third round (Fig. 3). The score achieved in the first round was 66 ± 39 points, that achieved in the second round was 82 ± 28 points, and that achieved in the third round was 89 ± 22 points (Fig. 4).

Subsequent ANOVA revealed that the participants increased in speed with each successive round of training (*p* < 0.001). The achieved score improved significantly between rounds 1 and 2 (*p =* 0.002) and between rounds 2 and 3 (*p =* 0.029).

Gaming experience had a significant impact on time (*p =* 0.039) and achieved score (*p =* 0.023). Age had no significant impact on time or score. Furthermore, medical specialization had no significant impact on score (*p* = 0.117) or time (*p* = 0.073). Subsequent post hoc tests showed only a significantly improved score in the third run for the emergency physician group vs. the group of certified nurses (99 vs. 78, *p <* 0.05). All other post hoc tests revealed no significant differences (Table 3).

**Table 3:** Achieved score and time for each run categorized by medical specialty

	Otorhinolaryngologists	Anaestesiologists	Emergency physicians	Certified nurses	*p* value
**N**	39	45	30	32	
**Score first run**					0.066
Mean ± SD (Median)	63 ± 40 (70)	72 ± 29 (80)	64 ± 44 (85)	63 ± 46 (60)	
**Score second run**					0.098
Mean ± SD (Median)	79 ± 30 (100)	84 ± 28 (100)	83 ± 27 (95)	83 ± 29 (80)	
**Score third run**					0.123
Mean ± SD (Median)	87 ± 23 (100)	94 ± 14 (100)	99 ± 5 (100)	78 ± 32 (80)	
**Time first run (s)**					0.454
Mean ± SD (Median)	84 ± 27 (83)	84 ± 25 (85)	95 ± 24 (97)	87 ± 31 (94)	
**Time second run (s)**					0.826
Mean ± SD (Median)	63 ± 27 (57)	60 ± 23 (56)	59 ± 24 (52)	65 ± 32 (53)	
**Time third run (s)**					0.882
Mean ± SD (Median)	49 ± 23 (46)	48 ± 19 (44)	45 ± 14 (42)	51 ± 30 (41)	

The Kendall-Tau correlation revealed no significant correlation between a subjective improvement in speed (Q6) and the time of the first round in the VR simulation (Kendall τ: -0.080, *p =* 0.204; Kendall τ second round: -0.015, *p =* 0.810; and Kendall τ third round: -0.077, *p =* 0.218). Comparable results were observed for the correlation between subjective improvement in the procedure (Q7) and the achieved score in the VR simulation. There was no significant correlation between the scores in the first (Kendall τ: 0.030, *p =* 0.663), second (Kendall τ: 0.052, *p =* 0.465) and third rounds (Kendall τ: 0.087, *p =* 0.239) and agreement with a subjective improvement in cricothyrotomy (Q7).

## Discussion

This prospective exploratory study is the first to decidedly evaluate the impact of a VR simulation of cricothyrotomy in different medical specialties in a training setting. Cricothyrotomy is a surgical intervention that offers the last opportunity to secure the airway in a “cannot intubate, cannot ventilate” situation. Fortunately, this procedure is very rare, which makes it difficult to perform it regularly and develop a routine.

The study results revealed a consistently positive evaluation of VR simulation for cricothyrotomy training. The evaluation of questions Q6 (I improved my speed during cricothyrotomy through VR simulation) and Q7 (I improved the way I performed a cricothyrotomy using VR simulation) represent the most important subjective items for the evaluation of the VR simulation, as both questions reflect the essential skills for a cricothyrotomy that must be acquired and refined during training. A total of 74% of the participants agreed that VR simulation helped them improve their speed during a cricothyroidotomy. Additionally, 87% of the participants reported that the VR simulation enhanced their subjective ability to perform a cricothyrotomy. The subjective improvement in speed could also be objectified by measuring the time required to complete the procedure. These subjective experiences during training may indicate that the created VR simulation can achieve relevant improvements in participants when performing a cricothyrotomy. However, it is important to note that the VR simulation is merely training an algorithm, reflecting Grade I and II on Kirkpatrick’s learning model [11] and that conclusions about real-life skills can only be drawn to a limited extent.

In addition, the majority of participants evaluated the VR simulation as intensive, which was intended to reflect the high-pressure situation during a cricothyrotomy. The participants rated the visual and acoustic signals (64.4%) as well as the time limit (83.6%) as factors that contributed to a higher intensity of the simulation. These elements also evoked the same impressions in the group of participants who had previously performed a cricothyrotomy in a real-life setting. The authors therefore assume that the simulation is feasible for reflecting a real-life high-pressure situation.

The survey findings indicated that the ORL, certified nurse and emergency physician groups perceived their competence to be enhanced following the simulation in comparison to their perceptions prior to the simulation. However, the anesthesiologists did not agree with this assessment. Furthermore, the VR simulation was evaluated as good overall by all the groups.

The consistently positive results could not be measured in a questionnaire-based study on the use of virtual reality in the further training of medical students [12]. This suggests that the use of VR is evaluated and accepted differently depending on the target group. The medical specialties involved in our study were selected on the basis of their involvement in airway management in daily practice. The consistently favorable answers in the questionnaire indicate that VR simulation is an effective method for introducing subjects to cricothyrotomy, regardless of their prior knowledge and level of involvement in critical airway management in their daily routine.

Previous studies have demonstrated the efficacy of simulation training in cricothyrotomy for both anesthetists [13] and medical students [14].

Numerous different simulation trainers for cricothyrotomy are described in the literature. In addition to 2D simulators on desktop computers that enable the procedure via haptic interface devices [14–17], the procedure is trained on simulation manikins on which other procedures, such as thoracostomy and pericardiocentesis, can be practiced [18]. Another frequently used model is the pig cadaver, which offers the possibility of dissection of the tissue surrounding the larynx and trachea. Even after the introduction of more modern simulation options, porcine cadavers continue to be used for learning cricothyrotomy [5, 18, 19] and were even found to be more effective than training with simulation manikins in a 2016 study from Takayesu et al. in terms of surgical accuracy [20]. This may be attributed to the fact that a replica often has a distinctive physical and behavioral profile in comparison to a natural material. The use of VR has been extensively documented by numerous authors, particularly within the domain of emergency medicine. In this context, VR is frequently employed as a tool for navigating complex decision-making processes and the subsequent outcomes, effectively simulating real-world scenarios. Participants in a study investigating CPR training in a VR-environment were even the opinion that the VR environment could potentially replace training with manikins entirely [21]. In the case of endoscopy and laparoscopy trainers, where the user interacts with authentic instrument handles and the camera image of the operating theatre on the monitor is replaced by a virtual reality environment, an actual learning gain at the skills level has been observed [22, 23].

Compared with the VR simulation investigated in this study, the financial costs associated with such models are considerably higher, and scalability is a significantly more challenging aspect. This is an important factor to consider if the goal is to provide emergency training to a large number of individuals, potentially in challenging contexts during crises or combat situations [24], with the highest possible number of repetitions of the procedure. On the other hand, training models with haptic feedback have an advantage in teaching the learner to perform the actions. This must be taken into account when choosing a cricothyrotomy training program.

The positive evaluation of the VR simulation for cricothyrotomy training with regard to questions 6 and 7 indicates its potential efficacy in facilitating the acquisition of the cricothyrotomy procedure algorithm. However, a limitation noted in comparison to traditional cricothyrotomy trainers is the absence of haptic feedback, which 89% of participants in a study by Qi et al. deemed useful [15]. While the lack of haptic feedback may limit the tactile realism of the training, it allows learners to focus on internalizing the cognitive and procedural steps of the intervention. This can reduce cognitive load, particularly during initial training stages, by emphasizing the timing and rationale of specific actions. Nonetheless, the importance of haptic feedback in manual interventions should not be underestimated. Studies involving endoscopy and laparoscopy trainers have demonstrated that incorporating a haptic feedback mechanism significantly enhances skill acquisition [22, 23, 25]. This suggests that while VR simulations can be effective in building procedural knowledge, they function best as a complementary tool alongside traditional haptic trainers in comprehensive training programs. Integrating haptic feedback into future VR simulations could bridge the gap between cognitive understanding and tactile execution, creating a more holistic learning experience. Despite the current limitation, the VR simulation was rated positively, underscoring the importance of other factors, such as the integration of visual and acoustic signals and time constraints, in simulating high-pressure scenarios. In the training environment, the scores achieved and the time required to perform the VR cricothyrotomy improved significantly and consistently across all groups over the course of the three sessions. These findings are consistent with those of other studies on cricothyrotomy trainers, which have also demonstrated improvements during the training process [14, 15]. The evaluation demonstrated only minimal differences between the groups in terms of the scores achieved and the time taken, indicating that the use of a VR simulation is an effective tool for teaching cricothyrotomy to all professional groups, regardless of their previous experience and basic medical training. In the third round, the emergency physicians outperformed the certified nurses in terms of the number of points achieved. This may be attributed to the unique experience and expertise of emergency physicians, who are regularly urged to adapt quickly to new situations. It is plausible that this provided them with an advantage in learning the procedure without errors, which led to higher scores.

To improve the efficacy of VR training, several gamification elements, including the use of a point system and a storyline, were implemented within our VR simulation. A total of 83.6% of the participants agreed that they were able to apply their theoretical knowledge to the VR simulation. The data indicate the potential for transferring computer programs for learning medical content. A survey conducted by Kron et al. revealed that 80% of the participants rated video games as a viable teaching method [26]. VR simulations provide standardized and safe environments for learning medical skills [25, 27, 28]. As our VR simulation is similar to a computer game, it seems unsurprising that our study revealed a positive, significant relationship between faster time and gaming experience as well as a better score and gaming experience.

A current review by Abbas highlights the problems of VR simulation research in medicine. Many studies on the topic show improvements in procedures and training settings, but there is currently no broad evidence that this is also reflected in an improvement in patient care [29].

### Limitations

The present study is limited by the lack of a control group for training and time. The present study does not perfectly reflect a real-life situation requiring cricothyrotomy. It is therefore possible that the results may not be entirely representative of an actual cricothyrotomy. Factors such as the stress level of the participants and haptic handling were not measured, and whether the training resulted in a long-lasting improvement in the procedures and the shorter time required for the procedure was not tested. The objective was to assess the feasibility of VR simulation training across various medical specialties without attempting to control the training effect in real life. One limitation is the fact that the participants completed the questionnaire after the cricothyrotomy. This may have introduced a bias with regard to the personal performance assessment before and after the training, resulting in a potentially exaggerated before-and-after effect. In addition, participation in the study was voluntary, which may have led to a disproportionate number of younger people and people with an affinity for VR volunteering as participants. This can lead to false positive approval ratings and an overestimation of the training effect in the actual target group.

## Conclusion

In conclusion, VR simulation appears to be a suitable tool to support members of various medical professions in learning the cricothyrotomy algorithm. The VR simulation was rated positively across all groups and demonstrated a training effect across different medical specialties. The utilization of VR in simulation-based surgical training has the potential to enhance educational outcomes in terms of knowledge acquisition and retention. However, there is currently insufficient evidence to directly improve patient outcomes. Further research is needed to assess the efficacy of the presented VR simulation for training cricothyrotomy in real-world scenarios.

## Electronic supplementary material

Below is the link to the electronic supplementary material.

Supplementary Material 1

## Data Availability

The datasets analyzed during the current study are available from the corresponding author upon reasonable request. Data of the used questionnaire is provided supplementary information files.

## Abbreviations

ORL: otorhinolarnygologist
VR: virtual reality
