# Supplementary material for: Virtual reality cricothyrotomy – a tool in medical emergency education throughout various disciplines
Source: BMC Med Educ. 2025 Feb 17;25:250. doi: 10.1186/s12909-025-06816-5 (PMC11831761; doi:10.1186/s12909-025-06816-5)
Supplement: Supplementary file 1 — Supplementary Material 1 [file 12909_2025_6816_MOESM1_ESM.docx]

Dear test subjects,

we would like to thank you for taking part in the study on the virtual reality simulation of a coniotomy. The study is being carried out by the Department of Otorhinolaryngology - Head and Neck Surgery Medical Centers of Freiburg and Tübingen. With the study, the authors would like to investigate the possibility of VR training for learning coniotomy in various medical disciplines. All data is collected anonymously. The first 10 questions ask for subjective impressions of the training; here we ask for honest answers about the experience of the training. Questions 11 - 15 are general questions about possible prior knowledge. In questions 16-18 we first ask for a personal assessment of your own coniotomy skills and the training as a whole; in this part school grades are used for grading.

| **Age:**   1. **Gender:** 2. **Medical specialty:** 3. **Years of working experience:** | | | | | | | | | | | | | | | | | | | | | | |
| --- | --- | --- | --- | --- | --- | --- | --- | --- | --- | --- | --- | --- | --- | --- | --- | --- | --- | --- | --- | --- | --- | --- |
|  | Very strongly disagree | | Strongly disagree | | | | Disagree | | | Neutral | | | Agree | | | Strongly agree | | Very strongly agree | | | N/A | |
| 1) I felt well prepared for the VR simulation. | 1 | | 2 | | | | 3 | | | 4 | | | 5 | | | 6 | | 7 | | | 0 | |
| 2) I was able to transfer my theoretical knowledge of performing a cricothyrotomy to the VR simulation. | 1 | | 2 | | | | 3 | | | 4 | | | 5 | | | 6 | | 7 | | | 0 | |
| 3) The distractors (gloves, disinfectants) influenced my approach. | 1 | | 2 | | | | 3 | | | 4 | | | 5 | | | 6 | | 7 | | | 0 | |
| 4) The visual and acoustic signals increased the intensity of the VR simulation. | 1 | | 2 | | | | 3 | | | 4 | | | 5 | | | 6 | | 7 | | | 0 | |
| 5) The time limit increased the intensity of the VR simulation. | 1 | | 2 | | | | 3 | | | 4 | | | 5 | | | 6 | | 7 | | | 0 | |
| 6) I improved my speed during a cricothyroidotomy through VR simulation. | 1 | | 2 | | | | 3 | | | 4 | | | 5 | | | 6 | | 7 | | | 0 | |
| 7) I improved the way I performed a cricothyrotomy using VR simulation. | 1 | | 2 | | | | 3 | | | 4 | | | 5 | | | 6 | | 7 | | | 0 | |
| 8) My expectations of the VR simulation were met. | 1 | | 2 | | | | 3 | | | 4 | | | 5 | | | 6 | | 7 | | | 0 | |
| 9) Recording the time and awarding points awakened my ambition. | 1 | | 2 | | | | 3 | | | 4 | | | 5 | | | 6 | | 7 | | | 0 | |
| 10) The feedback at the end of the VR simulation was helpful. | 1 | | 2 | | | | 3 | | | 4 | | | 5 | | | 6 | | 7 | | | 0 | |
| 11) Do you have gaming experience? | Yes | | | | | | | | | | | | No | | | | | | | | | |
| 12) I have experience in VR simulation. | Yes | | | | | | | | | | | | No | | | | | | | | | |
| 13) I play VR games regularly. | Yes | | | | | | | | | | | | No | | | | | | | | | |
| 14) I have had to do a cricothyrotomy before. | Yes | | | | | | | | | | | | No | | | | | | | | | |
| 15) If yes, was the cricothyrotomy successful? | Yes | | | | | | | | | | | | No | | | | | | | | | |
| 16) I value my competence in cricothyrotomy BEFORE the VR training. | 1 | | | | 2 | | | | 3 | | | | 4 | | | | 5 | | | 6 | | |
| 17) I value my competence in cricothyrotomy AFTER the VR training. | 1 | | | | 2 | | | | 3 | | | | 4 | | | | 5 | | | 6 | | |
| 18) Overall rating of the VR simulation (1-15) | 1 | 2 | | 3 | | 4 | | 5 | 6 | | 7 | 8 | | 9 | 10 | | 11 | 12 | 13 | | 14 | 15 |

**Table 4.** The adapted questionnaire is based on the Münster evaluation questionnaire “additional role-playing module” (4, 9).
